# The Effect of Ultraviolet Irradiation on the Physicochemical Properties of Poly(vinyl Chloride) Films Containing Organotin(IV) Complexes as Photostabilizers

**DOI:** 10.3390/molecules23020254

**Published:** 2018-01-28

**Authors:** Duaa Ghazi, Gamal A. El-Hiti, Emad Yousif, Dina S. Ahmed, Mohammad Hayal Alotaibi

**Affiliations:** 1 Department of Chemistry, College of Science, Al-Nahrain University, Baghdad 64021, Iraq; duaa.g934@gmail.com; 2 Cornea Research Chair, Department of Optometry, College of Applied Medical Sciences, King Saud University, P.O. Box 10219, Riyadh 11433, Saudi Arabia; 3 Department of Chemistry, College of Science, Tikrit University, Tikrit 34001, Iraq; dinasaadi86@gmail.com; 4 Center of Excellence in Integrated Nano-Systems, King Abdulaziz City for Science and Technology, P.O. Box 6086, Riyadh 11442, Saudi Arabia; mhhalotaibi@kacst.edu.sa

**Keywords:** poly(vinyl chloride), photodegradation, ciprofloxacin, organotin(IV) complexes, ultraviolet irradiation, scanning electron microscope, atomic force microscope

## Abstract

Three organotin(IV) complexes containing ciprofloxacin as a ligand (Ph_3_SnL, Me_2_SnL_2_ and Bu_2_SnL_2_; 0.5% by weight) were used as additives to inhibit the photodegradation of polyvinyl chloride films (40 µm thickness) upon irradiation with ultraviolet light (λ_max_ = 313 at a light intensity = 7.75 × 10^−7^ ein dm^−3^ S^−1^) at room temperature. The efficiency of organotin(IV) complexes as photostabilizers was determined by monitoring the changes in the weight, growth of specific functional groups (hydroxyl, carbonyl and carbene), viscosity, average molecular weight, chain scission and degree of deterioration of the polymeric films upon irradiation. The results obtained indicated that organotin(IV) complexes stabilized poly(vinyl chloride) and the dimethyltin(IV) complex was the most efficient additive. The surface morphologies of poly(vinyl chloride) films containing organotin(IV) complexes were examined using an atomic force microscope and scanning electron microscopy. These showed that the surface of polymeric films containing organotin(IV) complexes were smoother and less rough, compared to the surface of the blank films. Some mechanisms that explained the role of organotin(IV) complexes in poly(vinyl chloride) photostabilization process were proposed.

## 1. Introduction

The market for poly(vinyl chloride) (PVC) has grown significantly over the years due low production cost and its large versatility [1,2,3]. However, environmental factors such as sunlight, ultraviolet (UV) radiation, moisture, and high temperatures can lead to various chemical and physical changes in the PVC materials [4,5,6]. Ultraviolet radiation has deleterious effects on PVC such as the scissions of the polymeric chains that lead to the loss of mechanical properties such as strength, elasticity, and color changes [7]. In addition, it can lead to the softening of polymeric materials, surface cracking, loss of transparency, bleaching, and surface erosion [8,9].

A UV light of a wavelength longer than 190–220 nm is not expected to be absorbed by PVC since it only contains single bonds (C-C, C-H, and C-Cl) [10]. Commercially available PVC has various structural defects due to the presence of several other molecules in small quantities [11]. The structural defects due to the presence of photosensitive chromophores such as allylic chlorine due to random unsaturation, tertiary bonded chloride, and impurities within the PVC polymeric chains can lead to photodegradation. In addition, the steric hindrance within the monomeric units, i.e., the tacticity, could influence the degradation process [9]. Therefore, the evaluation of the changes in the PVC properties under service conditions is very important in order to find ways to improve the durability of PVC containing products [12,13]. The susceptibility of PVC polymeric materials to UV light (300–400 nm) has been investigated [14,15,16]. Various additives at low concentrations have been used to inhibit the photodegradation process of PVC. The most common additives used are organometallics including organotin complexes [17,18,19], organics [20,21,22], Schiff bases [23,24,25,26,27], and others [28,29,30,31,32,33].

As a continuation of our research in the field of polymers [34,35,36,37,38,39,40], we began to investigate the effects of tin-ciprofloxacin complexes as additives at low concentrations (0.5% by weight) and their role in the inhibition of photodegradation of PVC films, upon irradiation with UV light for long period. We now report success in this respect in which the photostabilization effect of such additives was found to be better than the previously reported organotin complexes of leucine [17], 2-[(2,3-dihydroxyphenyl)methylideneamino]benzenesulfonic acid [18], and furosemide [19].

## 2. Results and Discussions

### 2.1. Organotin(IV) Complexes

**Figure 1:**
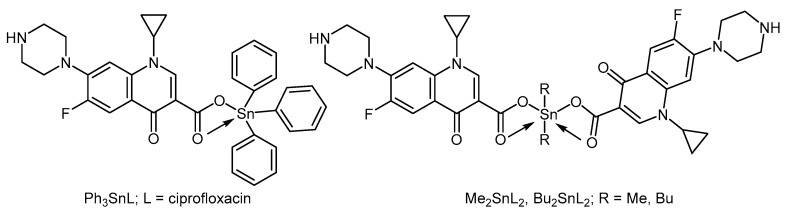
Organotin(IV)-ciprofloxacin complexes.

Three organotin-ciprofloxacin complexes (Ph_3_SnL, Me_2_SnL_2_ and Bu_2_SnL_2_; Figure 1) were synthesized as previously reported [41] in 52–67% yields and their colors range from white to pale yellow. The analytical and spectroscopic data for the synthesized complexes were consistent with the ones reported [41]. The synthesized organotin were varied in their structures in which diorganotin (Me_2_SnL_2_ and Bu_2_SnL_2_) and triorganotin (Ph_3_SnL) were used. Also, the substituents on the Sn were varied as small (Me) and bulky (Bu and Ph) groups.

### 2.2. Evaluation of PVC Photodegradation by Weight Loss

**Figure 2:**
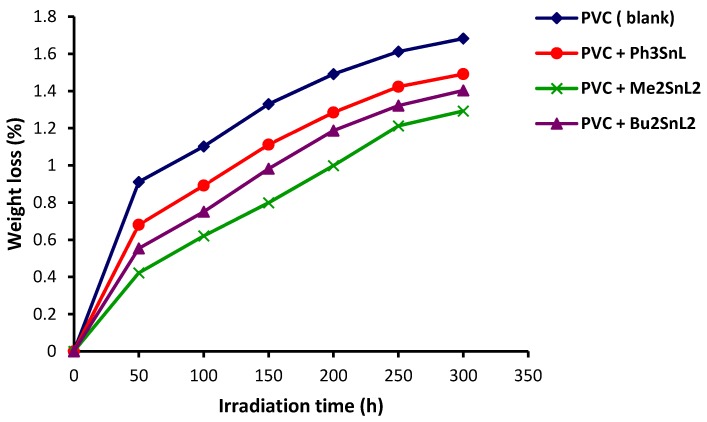
Changes in the weight loss (%) of poly(vinyl chloride) (PVC) versus time of irradiation.

Photodegradation of PVC produces low molecular weight fragments as well as hydrochloride (HCl) that lead to a weight loss [42]. The weight loss of PVC films (40 µm thickness), in the presence of organotin(IV) complexes (0.5% by weight) upon irradiation (300 h), was calculated and compared to that obtained for the blank PVC film. Figure 2 shows the changes in the PVC weight loss percentage at different time of irradiation (up to 300 h). Evidently, the PVC films containing organotin complexes show lower weight loss compared to that obtained for the blank PVC. Dimethyltin(IV) complex, Me_2_SnL_2_, shows the most efficient stabilization effect against PVC photodegradation. The dimethyltin(IV) complex is the least sterically hindered among the additives used and, therefore, it acts as a better primary photostabilizer compared to the others. Triphenyltin complex shows the least photostabilization effect presumably due to the presence of bulky phenyl groups.

### 2.3. Evaluation of PVC Photodegradation by FTIR Spectroscopy

**Figure 3:**
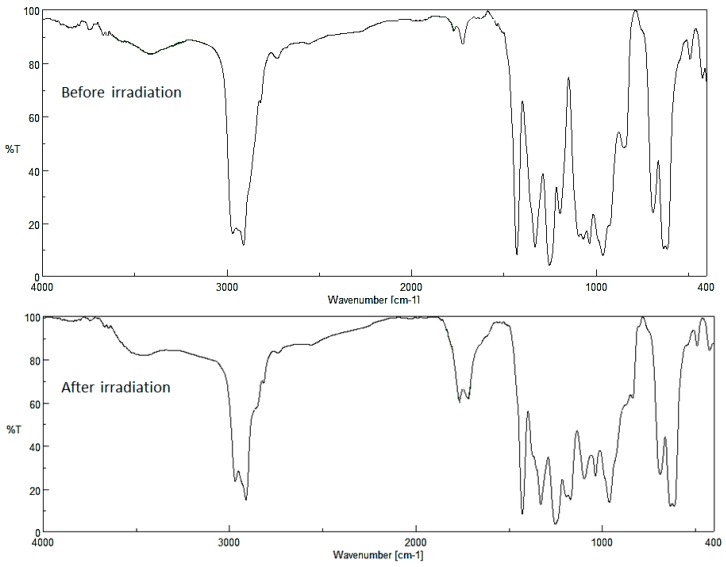
FTIR spectra of PVC film containing Me_2_SnL_2_ complex before and after irradiation.

Ultraviolet radiation of PVC films leads to the appearance of several functional group moieties, in the IR spectra, such as OH (3500 cm^−1^), C=O (1722 cm^–1^) and C=C (1602 cm^−1^) [43]. The FTIR spectra of PVC films containing Me_2_SnL_2_ complex before and after irradiation (300 h) are shown in Figure 3.

**Figure 4:**
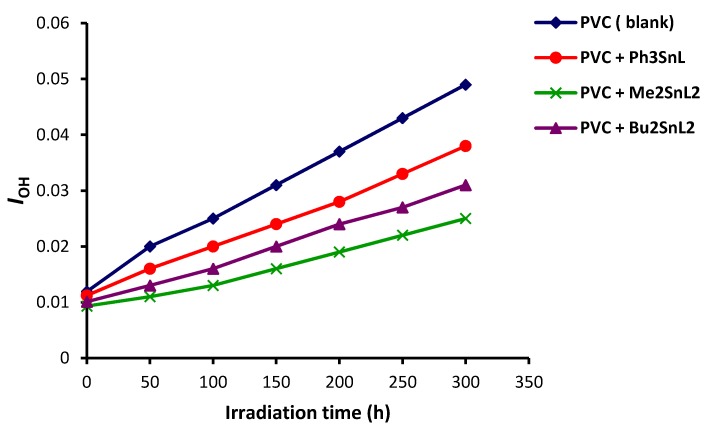
Changes in the *I*_OH_ index of PVC versus time of irradiation.

**Figure 5:**
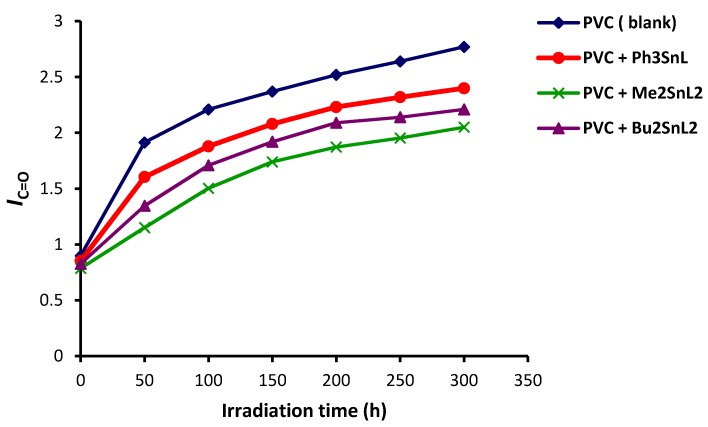
Changes in the *I*_C=O_ index of PVC versus time of irradiation.

**Figure 6:**
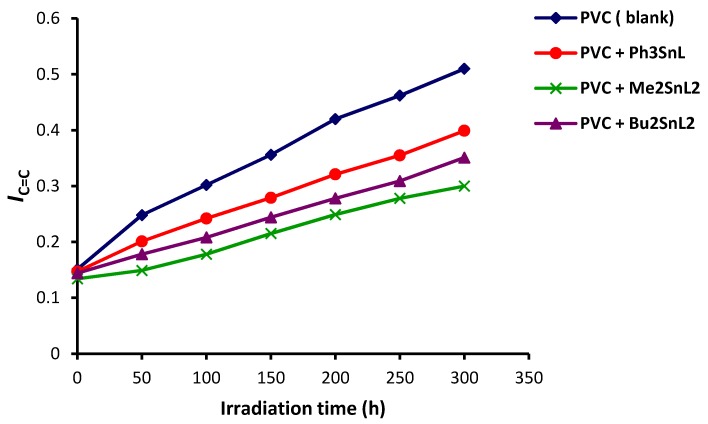
Changes in the *I*_C=C_ index of PVC versus time of irradiation.

The intensities of OH, C=O, and C=C peaks in the FTIR spectra were monitored for irradiated PVC with a UV light (λ max = 313 nm) and compared to that obtained for the reference peak (1328 cm^–1^) [44]. The *I*_OH_, *I*_C=O_, and *I*_C=C_ indices were calculated for the PVC films in the presence and absence of organotin additives (0.5% by weight) were calculated and plotted against time of irradiation (Figure 4, Figure 5 and Figure 6). The Me_2_SnL_2_ complex was the most effective photostabilizer among the ones used for PVC photostabilization followed by the Bu_2_SnL_2_ and Ph_3_SnL. The changes in the functional group indices were very noticeable for the blank PVC film compared to the ones containing organotin complexes. Clearly, such additives act as efficient photostabilizers to inhibit PVC photodegradation. Photo-oxidation of PVC leads mainly to chloroketone and ketone moieties along with alkene, hydroperoxide, and alcohol fragments [26]. Therefore, it was expected that the change in the *I*_C=O_ (Figure 5) would be larger compared to the changes in *I*_C=C_ (Figure 6).

### 2.4. Evaluation of PVC Photodegradation by Viscosity

**Figure 7:**
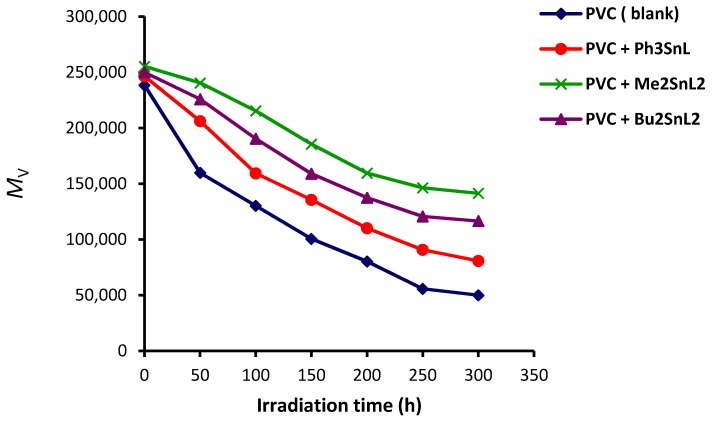
Changes in the ${\bar{M}}_{V}$ of PVC versus time of irradiation.

The exposure of PVC to UV light causes changes in the viscosity average molecular weight (${\bar{M}}_{V}$) [45]. The ${\bar{M}}_{V}$ for the PVC films upon irradiation was calculated at 25 °C in THF. The relationship between time of irradiation (h) and the changes obtained in ${\bar{M}}_{V}$ for PVC films (40 μm thickness) in the absence (blank) and presence of organotin(IV) complexes (0.5% by weight) was investigated (Figure 7). A sharp decrease in the ${\bar{M}}_{V}$ was observed for PVC (blank) in comparison to the PVC containing organotin as additives up to 250 h of irradiation [46]. The decrease in ${\bar{M}}_{V}$ was minimal for PVC containing Me_2_SnL_2_.

Some insoluble residues in THF were seen during the PVC photolysis process. The quantity of such residues could be used as an indicator for the average chain scission (*S*) due to the crosslinking and branching within the PVC polymeric chains [47]. Equation (1) can be used to calculate the *S* value from the viscosity average molecular weight at the initial time of irradiation (${\bar{M}}_{V,O}$) and at t time of irradiation (${\bar{M}}_{V,t}$).

(1) $$S={\bar{M}}_{V,O\;}/{\bar{M}}_{V,t}\;-1.$$

**Figure 8:**
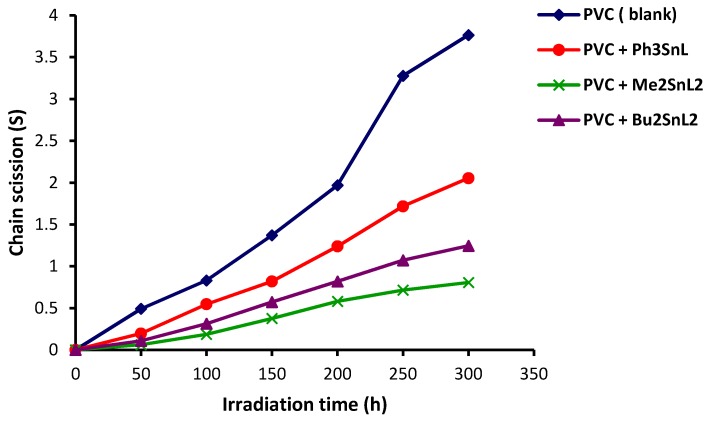
Changes in the chain scission (*S*) of PVC versus time of irradiation.

The changes in the *S* value for PVC films versus irradiation time (up to 300 h) is shown in Figure 8. There was a sharp increase in the *S* value for the blank PVC between an irradiation time of 100 and 300 h. The growth of the *S* value was less sharp for the PVC films that contain organotin(IV) complexes. Clearly, the additives used inhibit the photodegradation of PVC significantly. The increases in the *S* value was minimal when dimethyltin complex was present.

**Figure 9:**
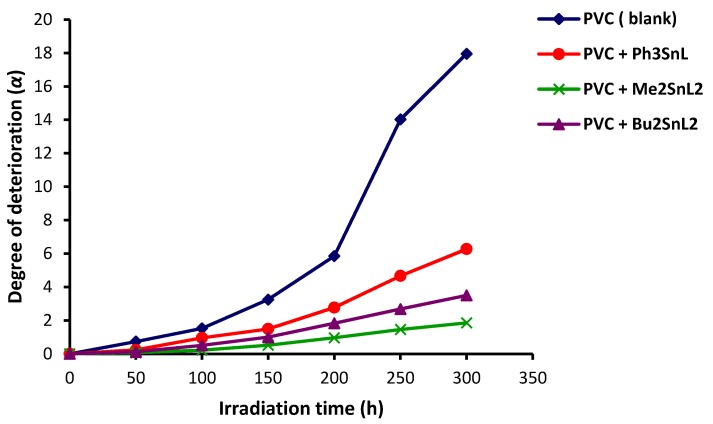
Changes in the degree of deterioration (α) of PVC versus time of irradiation.

At the initial stage of PVC photodegradation, randomly distributed weak bonds were rapidly broken. The degree of deterioration (α) which is a measure for PVC photodegradation can be calculated using Equation (2). The changes in the α values for PVC films containing photostabilizers on irradiation is represented in Figure 9.

(2) $$\alpha =m.S/{\bar{M}}_{V}$$

It can be seen that the α values for the irradiated PVC films containing organotin(IV) complexes were much less compared to the blank PVC. In the absence of additives, the *α* value increases with increasing time of irradiation. There was a sharp increase in the *α* values when the irradiation time increases from 150 to 300 h. On the other hand, the *α* value was minimal when dimethyltin(IV) complex was used as additive.

### 2.5. PVC Surface Morphological Study

#### 2.5.1. Microscopic Analysis

**Figure 10:**
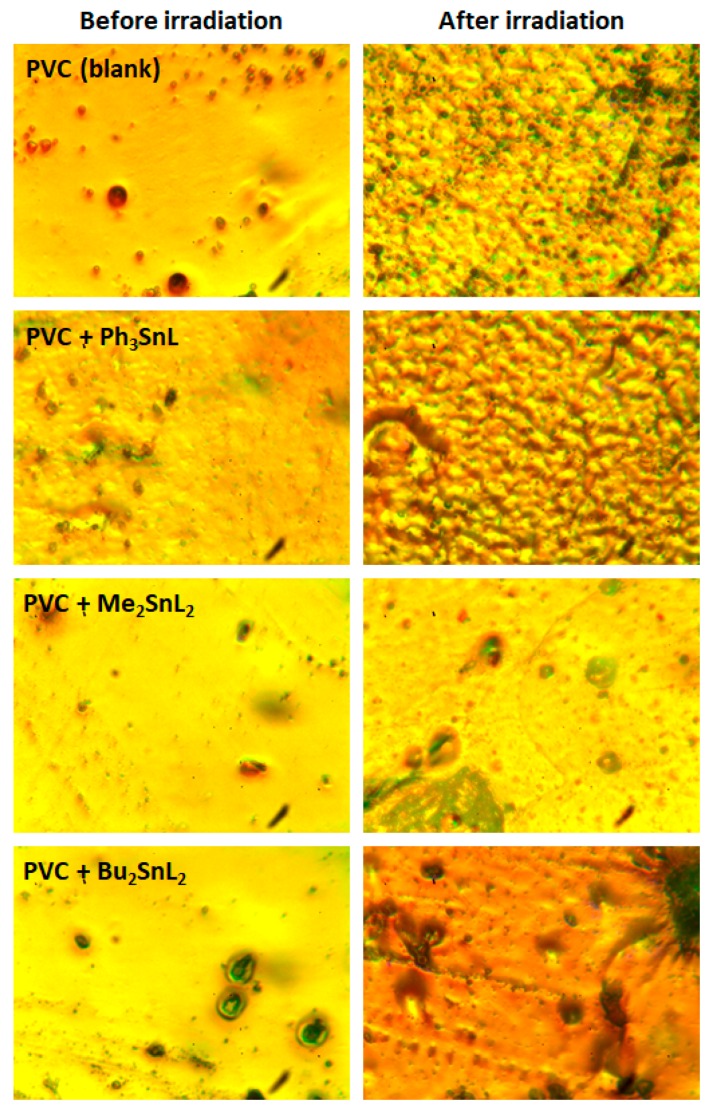
Microscope images of PVC films before and after irradiation.

The morphological study for the PVC films provides a clear picture about the crystalline case, surface irregularity, smoothness, and roughness of the surface. In addition, it provides a tool to detect the changes take place within the PVC surface due to photodecomposition [48]. The PVC surface images before and after 300 h of irradiation are shown in Figure 10.

It can be seen that the PVC surface before irradiation were smooth with no white spots present. While, the PVC surface after irradiation has a degree of surface damage in which cracks, holes, and grooves as well as change in the color possibly were noticeable as a result of photodegradation and evolution of volatile products (e.g., dehydrochlorination). The images for the irradiated PVC films containing organotin(IV) complexes show less cracks and white spots. The PVC surface was much smoother with fewer numbers of cracks and white spots when Me_2_SnL_2_ complex was used as the additive.

#### 2.5.2. Scanning Electron Microscope (SEM) Analysis

**Figure 11:**
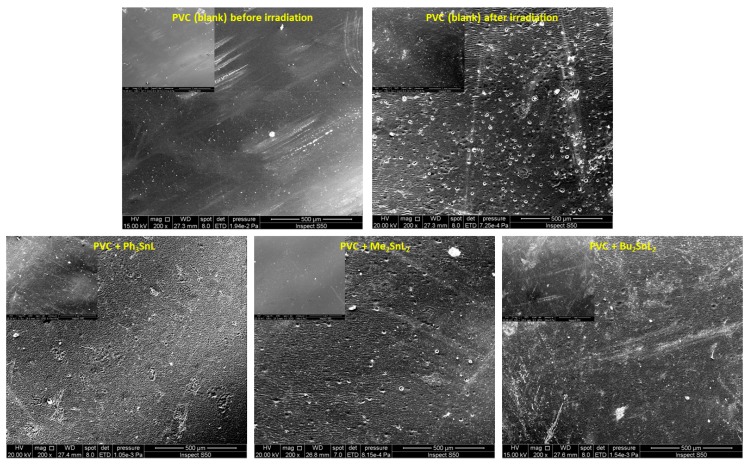
SEM images of PVC films.

The SEM examines the effect of UV irradiation on the surface morphology of PVC films [49]. The SEM images of PVC films are shown in Figure 11. The surface of the blank PVC film before irradiation was essentially smooth and neat. The PVC surface was damaged after 300 h of irradiation and the damage was much noticeable for the blank PVC compared to the ones containing the organotin additives. Also, the cracks were larger in length and depth compared to the non-radiated film. The formation of such cracks can be due to the chain crosslinking and evaluation of HCl and other volatile degradation molecules [50]. It was clear that the PVC film containing Me_2_SnL_2_ complex exhibits the least surface roughness and damage.

#### 2.5.3. Atomic Force Microscopy (AFM) Analysis

**Figure 12:**
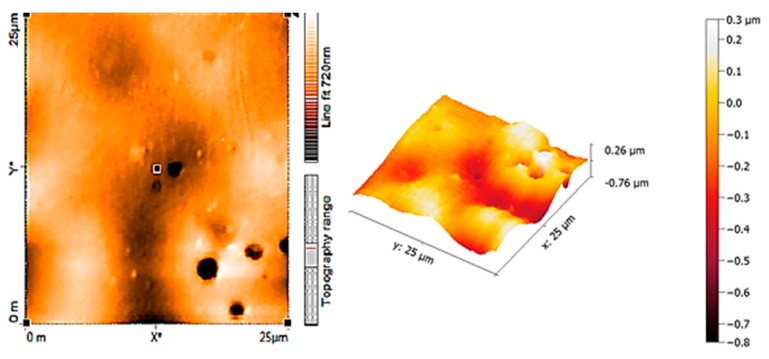
Atomic Force Microscopy (AFM) 2D and 3D images for PVC (blank) after irradiation.

**Figure 13:**
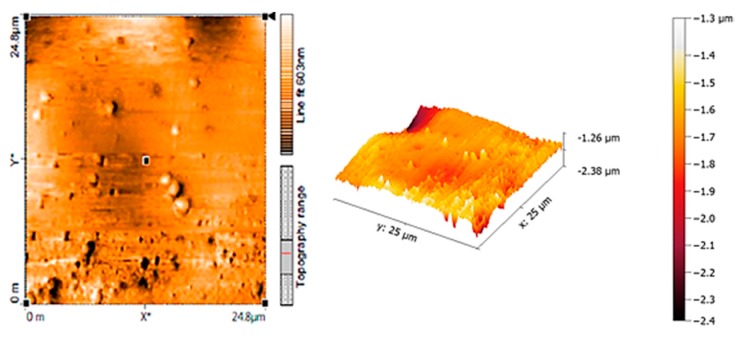
AFM 2D and 3D images for PVC containing Me_2_SnL_2_ after irradiation.

Atomic force microscopy (AFM) had been used to study the surface (area = 5.0 × 5.0 µm^2^) morphology of the PVC films after exposure to UV light for 300 h. The AFM 2D and 3D images for PVC (blank) and the one containing Me_2_SnL_2_ complex, as a photostabilizer, after irradiation are shown in Figure 12 and Figure 13, respectively. The PVC surface smoothness can be evaluated through the roughness factor (*R*q) [51]. High *R*q indicates dehydrochlorination and bond breaking that lead to rough surface [52,53]. Dehydrochlorination process normally takes place at high temperature [53]. The roughness factor was high (*R*q = 17.92) for the PVC (blank) compared to the one containing the Me_2_SnL_2_ complex (*R*q = 1.08) after 300 h of irradiation. Such result demonstrates the efficient use of dimethyltin complex to inhabit the PVC photodegradation.

### 2.6. Suggested Mechanisms for PVC Photostabilization by Organotin(IV) Complexes

**Scheme 1:**
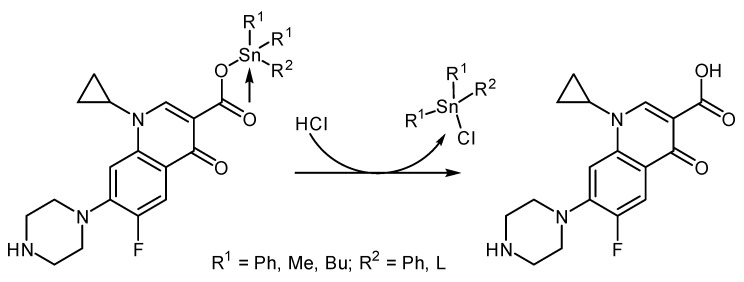
Organotin(IV) complexes as HCl scavengers.

The efficiency of di- and triorganotin(IV) complexes as PVC photostabilizers follow the order Me_2_SnL_2_ > Bu_2_SnL_2_ > Ph_3_SnL. The three organotin(IV) complexes used have reduced the PVC photodegradation significantly, but, the Me_2_SnL_2_ was the most effective one. Several mechanisms could be suggested to explain the photostabilization efficiency of these additives. Tin(IV) is a strong Lewis and, therefore, it acts as a good HCl scavenger (Scheme 1). Tin atoms can substitute the chlorine atoms within the PVC chains by the oxygen atoms of the carboxylate groups. Tin(IV) complexes can provide long-term PVC photostability by acting as secondary photostabilizers [29].

**Scheme 2:**
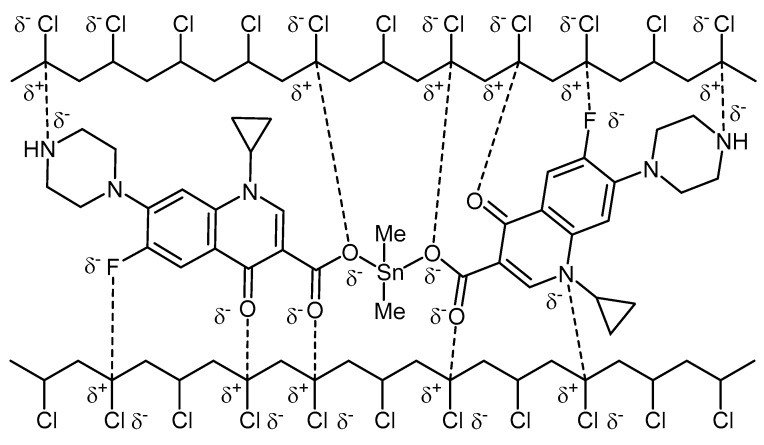
Organotin(IV) complexes as primary stabilizers.

The coordination between the polarized bonds within the organotin complexes and the C-Cl bonds within polymeric chains could inhibits the PVC photodegradation (Scheme 2). Organotin complex could act as primary photostabilizers by absorbing the light energy. Also, they help to release the energy of the PVC exited state, over time, to an energy level that is harmless to the polymeric materials [22,54]. Clearly, the steric effect within triphenyltin complex render such additive to be an efficient primary stabilizer.

**Scheme 3:**
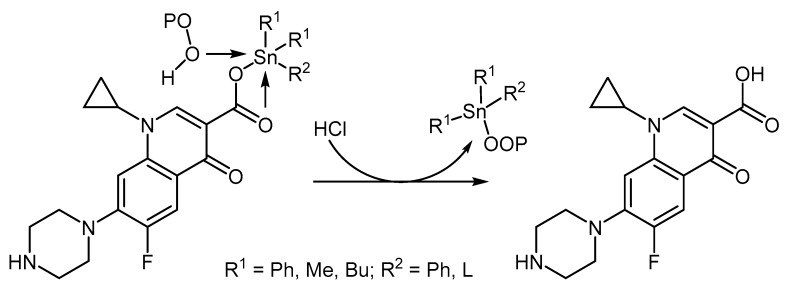
Organotin(IV) complexes as peroxide decomposers.

Organotin complexes could also act as peroxide decomposers to inhibit photodegradation of PVC. Photo-oxidation of PVC forms carbon radicals that lead to the production of peroxide radicals on reaction with oxygen [53]. Therefore, it is expected that tin complexes can decompose peroxides (e.g., hyrdroperoxides) and inhibit PVC photodegradation (Scheme 3) [55].

**Scheme 4:**
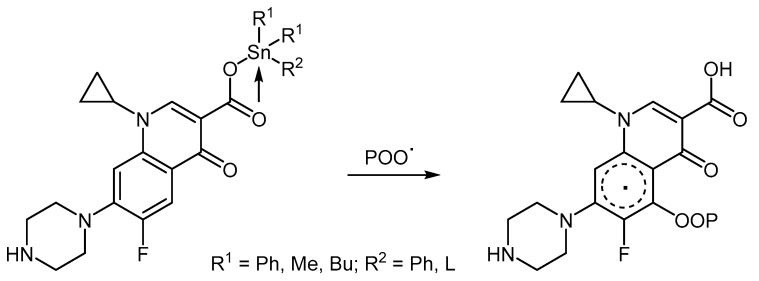
Organotin(IV) complexes as radical scavengers.

Organotin(IV) complexes could inhibit PVC photodegradation by acting as radical scavengers (Scheme 4). Complexation could take place between the chromophore (e.g., peroxide radical; POO·) and the additives to form un-reactive charge transfer complexes [55]. Also, organotin(IV) complexes can absorb UV light directly and the energy absorbed can be dissipated at harmless level to the polymeric chains possibly due to the resonance within the aromatic moieties [22].

## 3. Experimental

### 3.1. General

Ciprofloxacin, reagents, and solvents were supplied from Sigma-Aldrich Chemical Company (Gillingham, UK). PVC (K-value = 67, degree of polymerization = 800) was purchased from Petkim Petrokimya (Istanbul, Turkey). The Fourier transform infrared (FTIR) spectra of PVC films were recorded using a FTIR-8300 Shimadzu Spectrophotometer (Kyoto, Japan) at 400–4000 cm^−1^ (KBr disc). An accelerated weather-meter QUV tester (Philips, Saarbrücken, Germany) equipped with UV-B 313 lamps (λ_max_ = 313 nm and light intensity = 7.75 × 10^−7^ ein dm^−3^ s^−1^) was used to irradiate the PVC films (25 °C). The PVC surface morphology was inspected using Meiji Techno Microscope (Tokyo, Japan). The atomic force microscopy (AFM) images were recorded on Veeco instrument (Plainview, NY, USA). The scanning electron microscope (SEM) images of the PVC surface was recorded on the Veeco instrument (Veeco Instruments Inc., Plainview, New York, NY, USA) at an accelerating voltage of 15.00 kV.

### 3.2. Synthesis of Organotin(IV) Complexes

Triphenyltin(IV) complex containing ciprofloxacin (1-cyclopropyl-6-fluoro-4-oxo-7-piperazin-1-ylquinoline-3-carboxylic acid) as a ligand (Figure 1) was synthesized as previously reported [41]. The reaction of equimolar ratio of ciprofloxacin (L) and triphenyltin chloride (Ph_3_SnCl) in refluxing methanol for 6 h gave Ph_3_SnL complex as a white solid in 67%. Similarly, the reaction of excess ciprofloxacin (two mole equivalents) and dimethyltin dichloride (Me_2_SnCl_2_) or dibutyltin dichloride (Bu_2_SnCl_2_), in ethanol under reflux for 8 h, gave the corresponding Bu_2_SnL_2_ complex as a pale-yellow solid (52%) or Me_2_SnL_2_ complex as an off white solid (61%) [41].

### 3.3. Preparation of PVC Films

A solution of PVC (5 g) and organotin(IV) complexes (0.5% by weight) in tetrahydrofuran (THF; 100 mL) were stirred at 25 °C for 30 min. The mixture was casted onto glass plates and left at 25 °C for 24 h to ensure evaporation of residual of THF and the produced films were fixed.

### 3.4. Evaluation of PVC Photodegradation by Weight Loss

The photostabilization potency of organotin(IV) complexes as additives was evaluated by measuring the weight loss percentage within PVC films during irradiation using Equation (3), where, *W*_1_ and *W*_2_ are the PVC weight before and after irradiation, respectively [55]. The PVC weight was measured using Sartorius Lab–BL 219S electronic balance (Sartorius, Göttingen, Germany) with an accuracy of 0.0001 g.

(3) $$Weight\;loss\;\%\;=\left[\left(W_{1}-W_{2}\right)/W_{1}\right]\;\times \;100.$$

### 3.5. Evaluation of PVC Photodegradation by FTIR Spectroscopy

Photodegradation of PVC leads to the formation of a number of functional groups [56]. It has been reported that PVC photo-oxidation produces hydroxyl, carbonyl, and conjugated double bonds moieties [10,57,58]. The degree of photodegradation can be determined by monitoring the signals for such groups in the FTIR spectra (400–4000 cm^−1^) of PVC films upon irradiation. The changes in hydroxyl (*I*_OH_; 3500 cm^–1^), carbonyl (*I*_C=O_; 1722 cm^−1^), and polyene (*I*_C=C_; 1602 cm^−1^) indices were calculated and compared to a reference peak (1328 cm^−1^) [44]. The reference peak is assigned to the scissoring and bending of the CH_2_ groups in the FTIR spectra for PVC. Equation (4) was used to calculate the functional group index (*I*_S_) from the absorbance of the functional group (*A*_S_) and the reference peak (*A*_r_).

(4) Is=As/Ar.

### 3.6. Evaluation of PVC Photodegradation by Viscosity

Viscosity, [*η*], can be used to evaluate the changes in PVC average molecular weight (${\bar{M}}_{V}^{\alpha }$) using the Mark–Houwink relation shown in Equation (5), where, *α* and *K* are constants [59].

(5) $$\left[\eta \right]=K{\bar{M}}_{V}^{\alpha }.$$

The PVC molecular weight was calculated from the intrinsic viscosities using Equation (6).

(6) $$\left[\eta \right]=1.38\;\times \;10^{-4}\;{{\bar{M}}_{V}}^{0.77}$$

## 4. Conclusions

Three organotin(IV) complexes containing ciprofloxacin as a ligand have been used as photostabilizers to inhibit the photodegradation of PVC films, upon irradiation with ultraviolet light for a long period. Various methods such as the growth of certain functional groups in the infrared spectra, percentage of weight loss, variation in molecular weight, chain scission and degree of deterioration were used to determine the efficiency of organotin(IV) complexes. In addition, the atomic force and scanning electron microscopy were used to assess the surface morphology of poly(vinyl chloride) films. The organotin(IV) complexes used act as efficient photostabilizers and reduce the photodegradation rate of poly(vinyl chloride). It is believed that organotin(IV) complexes act as hydrogen chloride scavengers, peroxide decomposers, free radical scavengers and UV absorbers. For future research using organotin complexes as PVC photostabilizers, the possible leakage of tin should be assessed in order to allow the long-term use of such additives.
